# Integrated transcriptomic and metabolomic analyses reveal the effects of callose deposition and multihormone signal transduction pathways on the tea plant-*Colletotrichum camelliae* interaction

**DOI:** 10.1038/s41598-020-69729-x

**Published:** 2020-07-30

**Authors:** Qinhua Lu, Yuchun Wang, Fei Xiong, Xinyuan Hao, Xinzhong Zhang, Nana Li, Lu Wang, Jianming Zeng, Yajun Yang, Xinchao Wang

**Affiliations:** 1 Tea Research Institute of Chinese Academy of Agricultural Sciences, Hangzhou, China; 2 Tea Research Institute, Nanjing Agricultural University, Nanjing, China

**Keywords:** Microbiology, Fungi

## Abstract

*Colletotrichum* infects diverse hosts, including tea plants, and can lead to crop failure. Numerous studies have reported that biological processes are involved in the resistance of tea plants to *Colletotrichum* spp. However, the molecular and biochemical responses in the host during this interaction are unclear. Cuttings of the tea cultivar Longjing 43 (LJ43) were inoculated with a conidial suspension of *Colletotrichum camelliae*, and water-sprayed cuttings were used as controls. In total, 10,592 differentially expressed genes (DEGs) were identified from the transcriptomic data of the tea plants and were significantly enriched in callose deposition and the biosynthesis of various phytohormones. Subsequently, 3,555 mass spectra peaks were obtained by LC–MS detection in the negative ion mode, and 27, 18 and 81 differentially expressed metabolites (DEMs) were identified in the tea leaves at 12 hpi, 24 hpi and 72 hpi, respectively. The metabolomic analysis also revealed that the levels of the precursors and intermediate products of jasmonic acid (JA) and indole-3-acetate (IAA) biosynthesis were significantly increased during the interaction, especially when the symptoms became apparent. In conclusion, we suggest that callose deposition and various phytohormone signaling systems play important roles in the tea plant-*C. camelliae* interaction.

## Introduction

Tea plant (*Camellia sinensis* (L.) O. Kuntze) is an important economic crop in China. Its fresh shoots and leaves contain abundant inclusions and are used as raw materials of tea, which is popular among many people. Tea leaves are easily attacked by pathogens, leading to tree wilting and crop failure. There are many pathogens that attack tea plants, such as *Colletotrichum* spp., *Pestalotiopsis* spp., and *Discula theae-sinensis*^1–3^, of which *Colletotrichum* is the most important pathogenic genus. *Colletotrichum* species cause disease in an extremely wide range of hosts and live as endophytes in plants^4^. To date, 17 species of *Colletotrichum* have been reported to infect tea plants in China^5^. Previous studies have suggested that *Colletotrichum camelliae* is a host-specific taxon occurring on *Camellia*^6^. *C. camelliae* is regarded as the dominant species in *Ca. sinensis* and has high virulence^2,5^.

Most *Colletotrichum* species are hemibiotrophs that initially develop biotrophic hyphae inside a living host, which later transition to necrotrophic secondary mycelia^7,8^. The progression of *Colletotrichum* growth in host plants includes germ tube growth, appressoria development and sporulation. When a spore lands on the surface of a host, it rapidly germinates and adapts to its environment. Then, the initiation of appressorium development is activated when the fungus perceives surface hardness^9^. After the transition to the necrotrophic stage, secondary hyphae kill the host cell and initiate sporulation to start a new infection cycle^10,11^. As an important filamentous plant pathogen, *Colletotrichum* and its model plant hosts, such as *C. higginsianum*-*Arabidopsis thaliana* and *C. orbiculare*-*Nicotiana benthamiana*, have been studied in great detail to illuminate the mechanism underlying host–pathogen interactions^7,12^.

To prevent attack from pathogens, plants apply several lines of defense in immunity^13^. Phytohormones play an important role in the signaling system and trigger the expression of various defense-responsive genes^14^. Salicylic acid (SA), jasmonic acid (JA) and ethylene (ET) are important components in the response to microbial attack. The JA content is low in unstressed plants but accumulates in plant tissues treated with pathogen-associated molecular patterns (PAMPs)^14^. JA is synthetized from the oxylipin pathway through oxidation, and its precursors include linoleic acid, linolenic acid and roughanic acid^15,16^. The ET signaling system is a vital component of the plant innate immunity system^17^. Methionine is a precursor of ET. Similar to JA, ET biosynthesis is triggered by the PAMPs that also enhance the expression of ET biosynthesis genes^14,18^.

Plant defense responses involve callose deposition. Microbial infection could induce callose depositions on the plant surface, which form beneath infection sites to defend against penetration^19,20^. Callose depositions are able to prevent pathogen attacks^21,22^. In the interaction between lettuce and *Plasmopara lactucae-radicis*, lettuce resists attack by developing callose deposits around the haustoria^23^. However, the role of callose deposition in the tea plant-*C. camelliae* pathosystem remains unclear.

In recent years, few studies have focused on the interaction between tea plants and *Colletotrichum.* Wang et al. found that key genes involved in (−)-epigallocatechin-3-gallate, (+)-catechin, and caffeine biosynthesis were induced when *C. fructicola* colonized tea plants. According to the results of an antifungal bioassay, the authors suggested that the key secondary metabolites of tea plants have antifungal effects on *C. fructicola* growth^24^. Shi et al. compared healthy tea leaves and diseased tea leaves infected with *Colletotrichum* in the field, and their results showed that the differentially expressed genes (DEGs) were highly enriched in oxidation reduction processes, cell wall reinforcement, and plant hormone signal transduction, and SA was a key compound in the response of tea plants to *Colletotrichum*^25^. Elevated CO_2_ can increase the susceptibility of tea to *C. gloeosporioides* by reducing the endogenous caffeine content in tea plants, which is related to the JA-independent lipoxygenase (LOX) pathway in tea^26^. Although *C. camelliae* infection in tea plants causes a serious tea plant disease that leads to brown scabs^2,6^, research on the interaction between tea plants and *C. camelliae* is rather limited.

In our previous studies, we identified that *C. camelliae* was pathogenic to the tea plant cultivar Longjing 43 (LJ43)^2^. Using transcriptional analysis, we found that hypersensitive cell death and hydrogen peroxide play crucial roles in the resistance of tea plants to this fungus^27^. In this study, to determine the interaction of tea plants and *C. camelliae* during the pathogenetic process. We integrated transcriptome and metabolome analyses to identify the potential mechanism of the tea plant-*C. camelliae* interaction from fungal colonization to the necrotic phase.

## Results

### Phenotypic characterization of infected and mock-treated tea leaves

**Figure 1:**
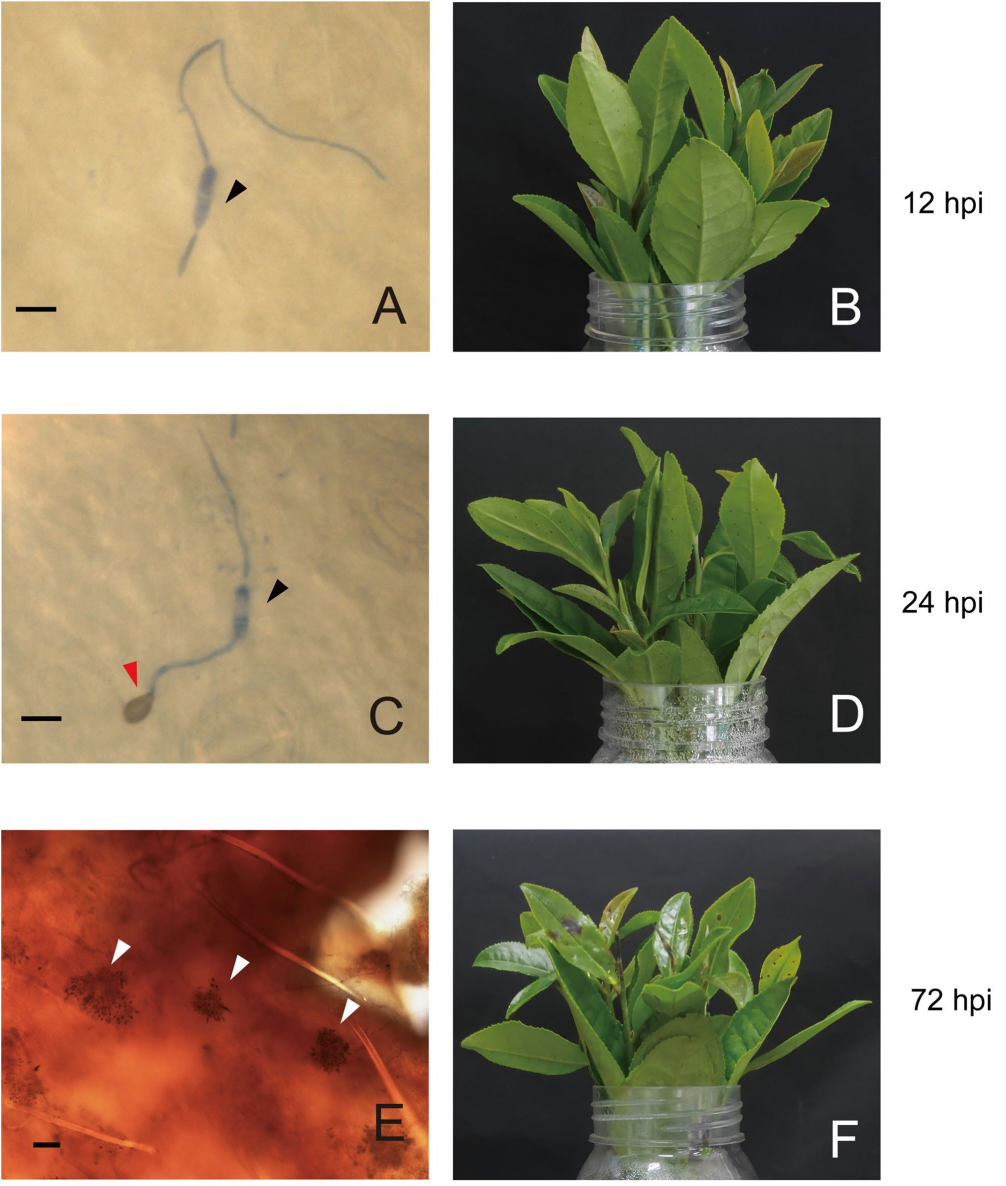
Phenotypic characterization of the tea plant-*C. camelliae* interaction and the expression profile of the fungus. (**A**,**C**,**E**) show the fungal form; (**B**,**D**,**F**) show the tea plant form. The black triangle indicates conidium, the red triangle indicates appressorium, and the white triangle indicates acervulus. Scale bar = 10 μm in (**A**,**C**); scale bar = 50 μm in (**E**).

Wounded leaves of LJ43 cuttings were inoculated with a *C. camelliae* spore suspension. Leaf appearance and fungal development were observed at 12, 24 and 72 hpi. At 12 hpi, germ tubes had formed, and no disease spots were observed on the surface of the tea leaves (Fig. 1A,B). At 24 hpi, tea leaves had not yet undergone obvious changes, and the fungus developed appressoria (Fig. 1C,D), which are effective infection structures and can secrete effectors to suppress host immunity^18^. As time progressed, scabs enlarged gradually, and the fungus initiated sporulation to facilitate the spread of disease (Fig. 1E,F). Compared to the control treatment, the leaves treated with *C. camelliae* blackened and even fell off the cutting.

### Transcription analysis of tea leaves during the plant–fungal interaction

To reveal the gene expression pattern of tea leaves in response to the fungus, samples were collected for transcriptome sequencing. The *Camellia sinensis* var*. sinensis* genome was used as a reference to map the transcriptome. A total of 936,786,400 high-quality clean reads were generated (Supplementary Table S1). Paired-end clean reads were aligned to the reference genome (RG) (Supplementary Table S1). The average alignment rates of the infected tea leaves were 90.86%, 90.76% and 87.75% in the 12, 24, and 72 hpi treatments, respectively. The average Q20 and Q30 of infected tea leaves respectively were 97.45 and 92.95, 97.24 and 92.50, 97.25 and 92.54 at 12 hpi, 24 hpi and 72 hpi. Based on the genes aligned with the RG and novel genes, 59,313 genes were identified, of which 56,134 were annotated with TAIR10 according to the significant hits (e-value < 1e^−5^).

**Figure 2:**
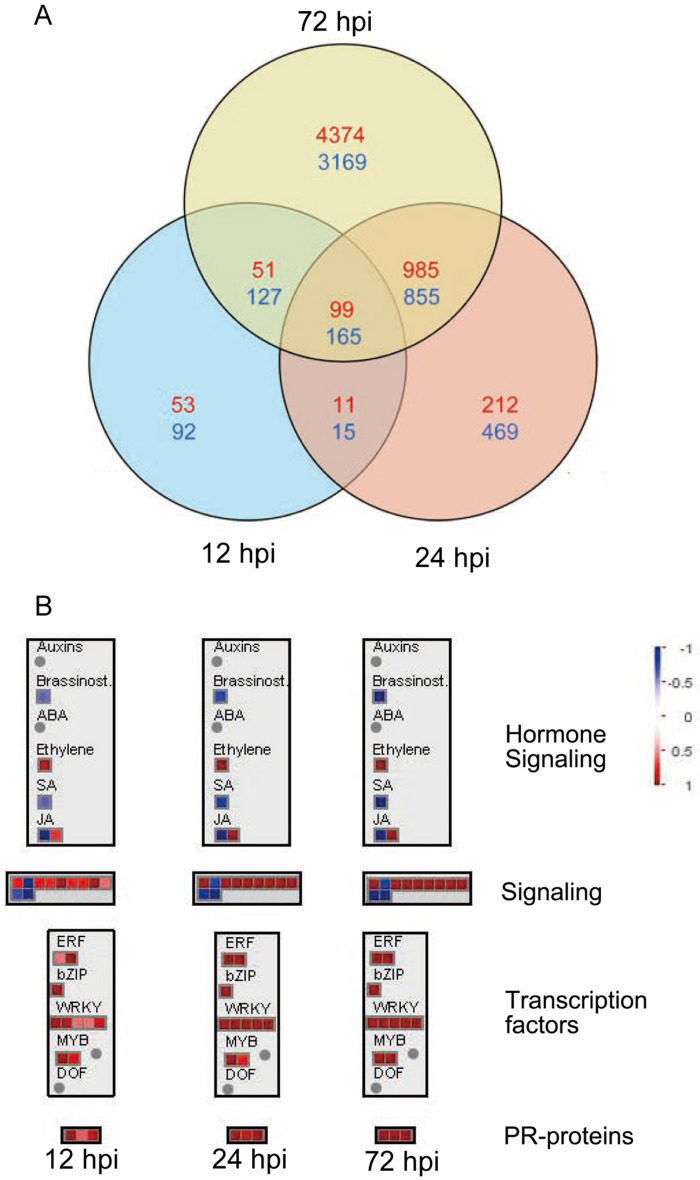
Analysis of the RNA-seq data. (**A**) Venn diagram showing the number of DEGs (P < 0.05, fold change ≥ 1.5). The upregulated genes are shown in red, and the downregulated genes are shown in blue. The color scale indicates the log_2_fold change in the infected tea leaves relative to the mock-treated tea leaves. (**B**) Schematic of the shared DEGs at three time points using the MapMan visualization platform.

In this study, a total of 10,592 DEGs were identified at three different time points when the infected and mock-treated tea leaves were compared. Detailed information of the DEGs is presented in Supplementary Table S2. At 12 hpi, 399 DEGs were downregulated and 214 were upregulated. At 24 hpi, 1504 DEGs were downregulated and 1,307 were upregulated. In addition, the number of DEGs was increased with expanded disease spots. At 72 hpi, 4,316 and 5,509 DEGs were downregulated and upregulated, respectively (Supplementary Table S2). There were 99 and 165 continuously upregulated and downregulated DEGs in response to pathogen infection, respectively (Fig. 2A).

### Defense-related responses induced in the tea plant upon *C. camelliae* infection

The common DEGs at all three time points were analyzed by MapMan (Fig. 2B). JA and ET showed a positive response, while SA and brassinosteroids showed a negative response in the hormone signaling module. Pathogenesis-related (PR) proteins and 80% of the transcription factors (TFs) in the MapMan visualization, including ERF, bZIP, WRKY, and MYB, were upregulated. In the signaling module, the upregulated genes included leucine-rich repeat (LRR) family proteins and calmodulin-binding proteins, and the downregulated genes included phytochrome defective E.

**Figure 3:**
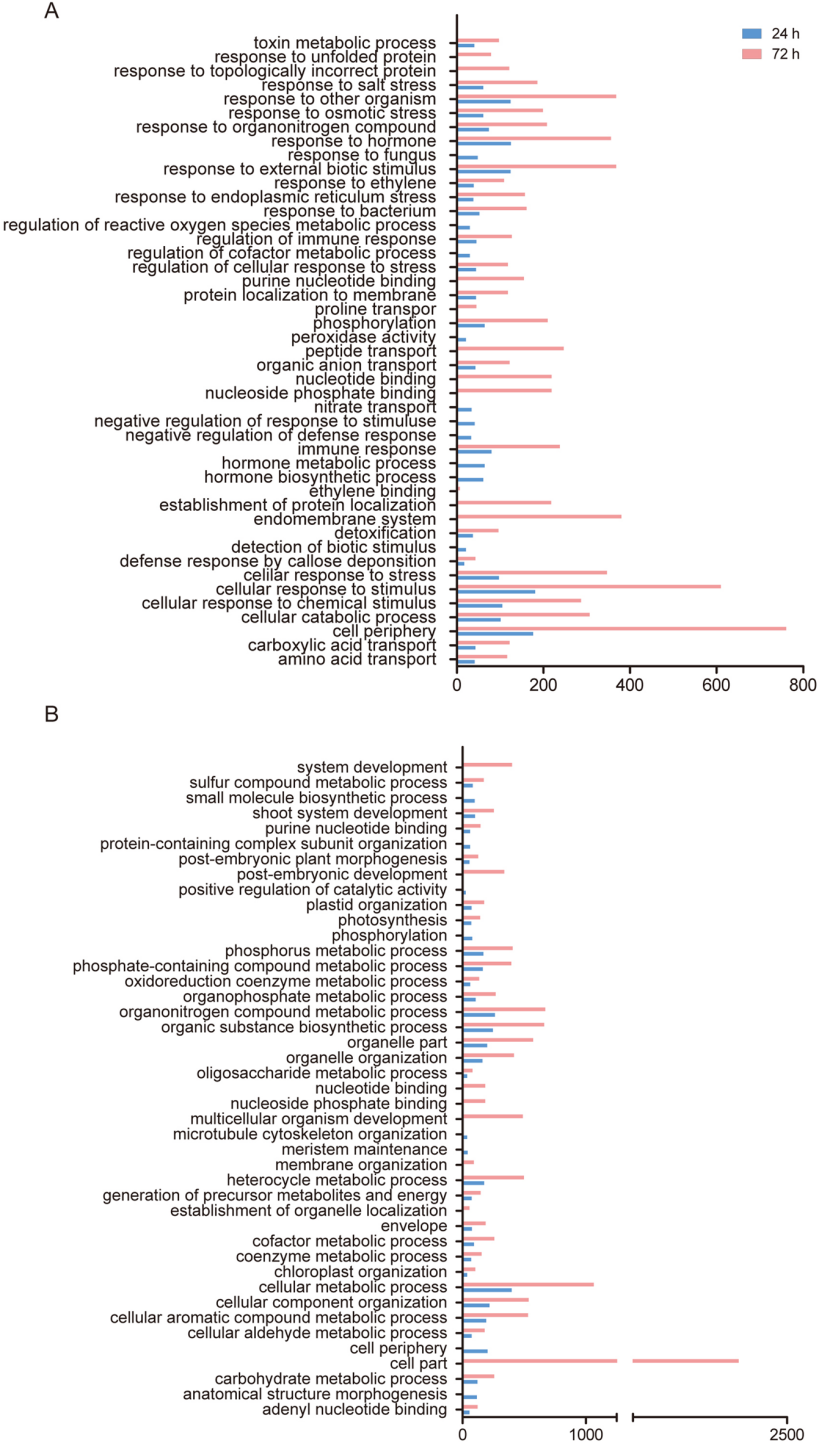
GO functional classification of up- and down-regulated DEGs at 24 h and 72 h. (**A**) GO functional classification of upregulated DEGs. (**B**) GO functional classification of downregulated DEGs.

In addition, the DEGs were annotated according to the GO database. The GO analysis indicated that all upregulated genes (5,785) and downregulated genes (4,892) were enriched in 178 and 244 terms, respectively (Supplementary Table S3). To determine the main pathways involved in different postincubation time points, we chose the top 35 pathways based on the p-value and ratio of the number in the input to the number in the reference (Fig. 3, Supplementary Table S4). We found that the upregulated sets were involved in multiple terms related to biotic stimulus.

**Figure 4:**
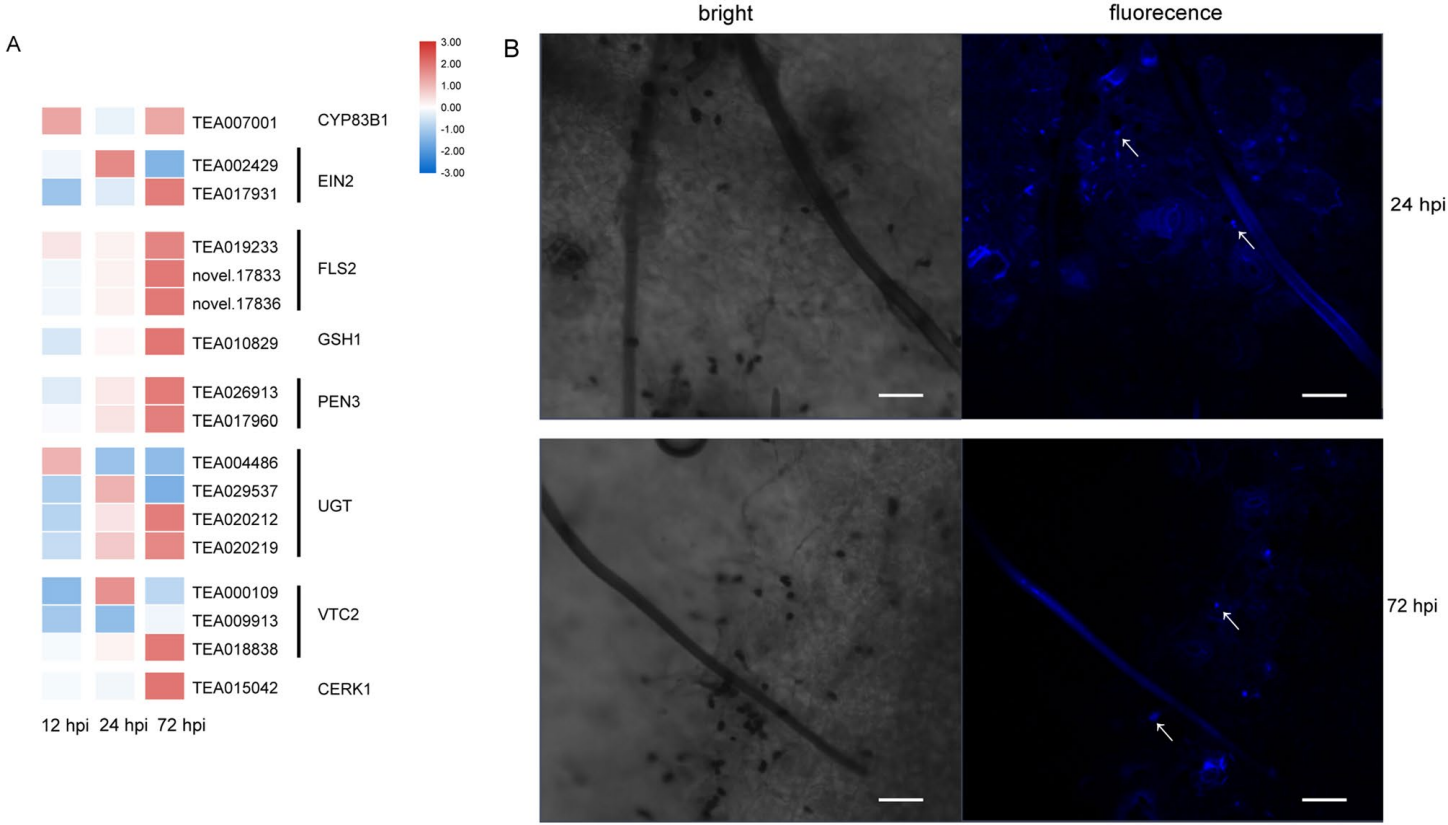
Callose deposition in *C. camelliae*-infected tea plants. (**A**) Expression heat map of the involved genes. (**B**) Aniline blue callose staining of tea plant leaves inoculated with *C. camelliae* at 24 hpi and 72 hpi. Scale bar = 50 μm.

One of the earliest cellular responses involved in the plant-pathogen interaction was peroxidase activity. At 12 hpi and 24 hpi, genes encoding enzymes involved in peroxidase activity, such as peroxidase superfamily proteins and glutathione peroxidase, were induced (Supplementary Table S4). Several genes associated with callose deposition were initially induced when the fungi developed infection structures, and the GO analysis indicated that the defense response by callose deposition (GO:0052542) was enriched (Supplementary Table S4). As shown in Fig. 1C,* C. camelliae* differentiated appressoria at 24 hpi. The callose deposition revealed by aniline blue staining was evident at infection sites at 24 hpi and 72 hpi (Fig. 4B). As shown in the heat map, most of the related genes began to be upregulated at 24 hpi when appressoria developed. As time progressed, the degree of gene expression and number of upregulated genes increased at 72 hpi. *flagellin-sensing 2 (FLS2*), *glutamate-cysteine ligase 1* (*GSH1*) and *penetration 3* (*PEN3*), which are associated with callose deposition, were upregulated consecutively during the interaction (Fig. 4A). These results suggest that tea plants applied physical mechanisms, such as cell wall reinforcement, in response to pathogen colonization.

**Figure 5:**
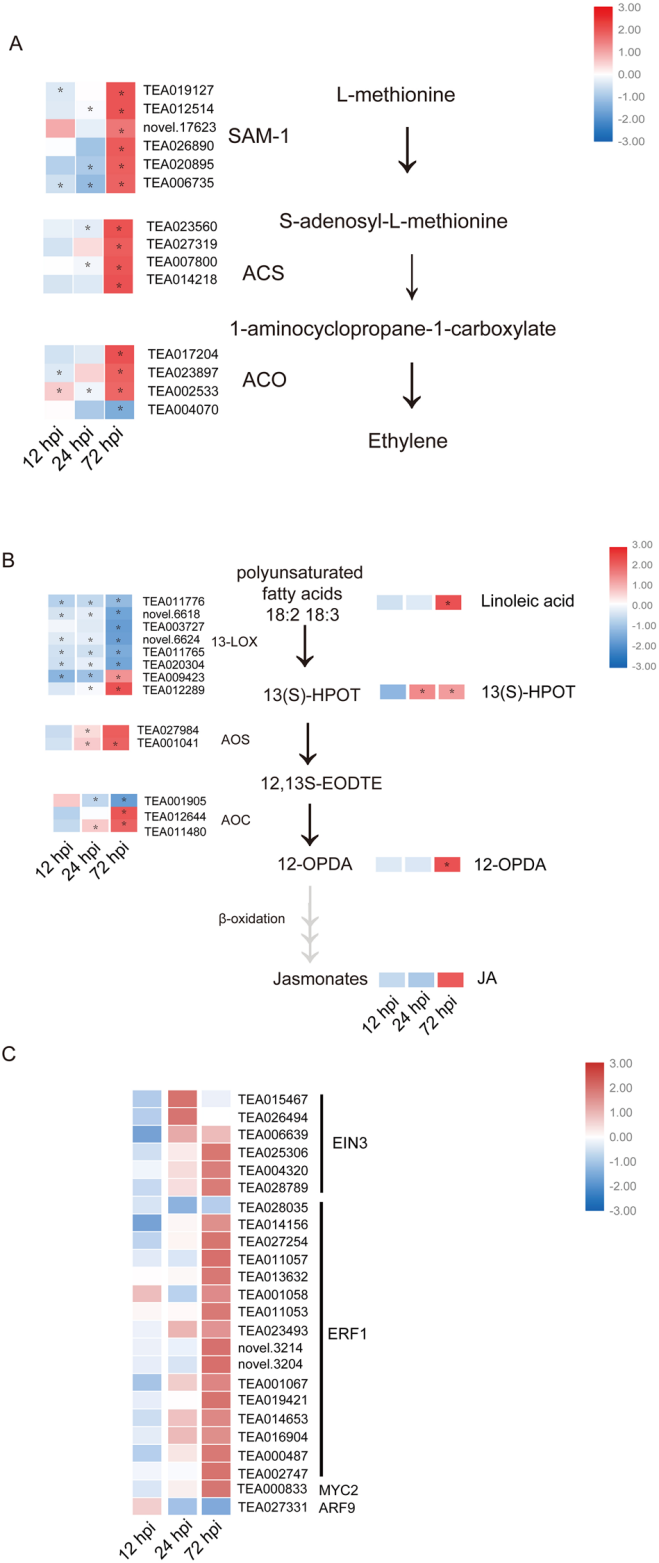
Multiple hormones are involved in the tea plant and *C. camelliae* interaction. (**A**) Heat maps of the expression of ET biosynthesis genes. (**B**) Heat maps of gene expression and the secondary metabolite value of JA biosynthesis. (**C**) Heat map showing TF expression related to the JA, ET and IAA pathways. *Indicates a statistically significant difference (P < 0.05) between the *C. camellia*-inoculated and mock-inoculated samples.

DEGs related to the response to hormones, such as ET, JA and indole-3-acetate (IAA), especially in the late stage of interaction, were upregulated. This suggested that hormones were important in the interaction between tea plants and *C. camelliae*. Almost all *S-adenosyl-l-methionine (SAM-1)*, *1-aminocyclopropane-1-carboxylic acid (ACC) synthase (ACS)* and *ACC oxidase (ACO)* transcripts, which are key genes for ET biosynthesis, were upregulated at 72 hpi. Several transcripts encoding ACO (TEA023897 and TEA002533) had a fold change of approximately 69 and 117, and genes encoding another key enzyme, ACS (TEA023560, TEA027319 and TEA007800), were upregulated approximately 20 times (Fig. 5A). ET-responsive TFs, such as ERF1 and EIN3, were significantly upregulated at 72 hpi (Fig. 5C). Other genes involved in the synthesis and signaling of JA and IAA showed changes in transcript levels following inoculation, suggesting hormonal interplay. Two transcripts encoding LOX, allene oxide synthase (AOS) and allene oxide cyclase (AOC), which are involved in JA biosynthesis, were also upregulated at 72 hpi (Fig. 5B). MYC2, which is related to JA signaling, changed significantly by 3.4-fold (Fig. 5C). Four transcripts encoding indole acetaldoxime dehydratase, which is involved in IAA synthesis, were differentially regulated (Supplementary Table S2). The TF ARF9 was significantly downregulated (Fig. 5C).

### Metabolomic analysis of infected tea leaves during the plant–fungal interaction

**Figure 6:**
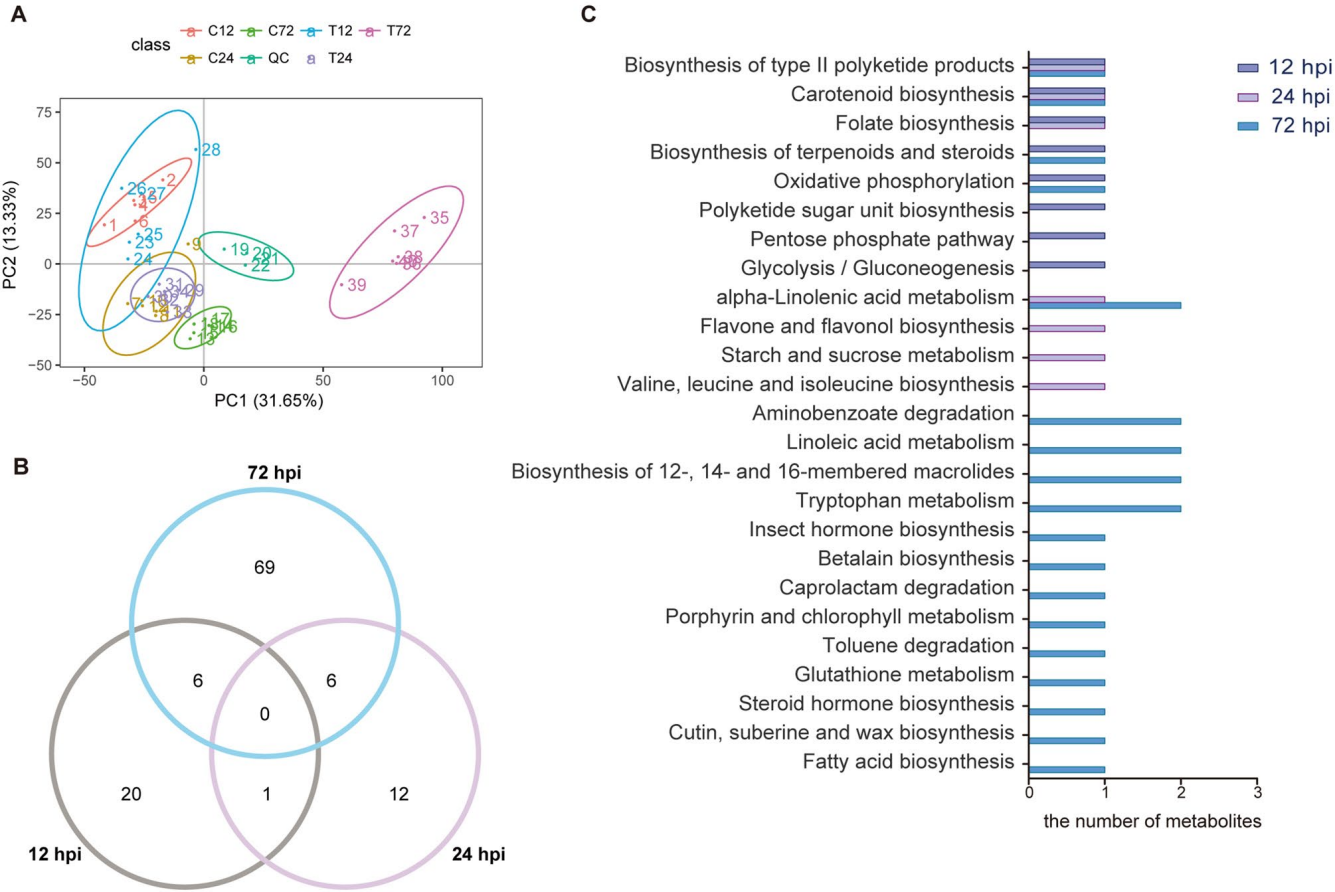
Analysis of the metabolomics data. (**A**) Principal component analysis (PCA) displaying the biological variations among the samples. (**B**) Venn diagram showing the number of DEMs (P < 0.05, fold change ≥ 1.5 and VIP ≥ 1). (**C**) KEGG functional annotation of the tea plant metabolome.

The metabolomic profiling of the tea plants was carried out using a Vanquish UHPLC LC–MS/MS system to characterize the global metabolome changes in the infected tea leaves relative to those in the control groups. The biological variability within replicates was analyzed by principal component analysis (PCA) (Fig. 6A). The first and second principal components (PC1 and PC2) explained 44.98% of the variation in gene expression. The first principal component separated the 72-h-treated versus untreated samples and samples from other time points. However, the samples from 12 and 24 hpi had substantial overlap in the PCA plot, suggesting that these samples were similar at the metabolome level.

This system detected 3,555 mass spectra peaks, 713 of which were annotated by KEGG. In this study, the differentially expressed metabolites (DEMs) were identified based on the following standards: |fold change|≥ 1.5, P-value < 0.05 and VIP ≥ 1 (Supplementary Table S5). We obtained 27, 18 and 81 DEMs at 12 hpi, 24 hpi and 72 hpi, respectively (Fig. 6B), and they included gluconic acid, fatty acid, amino acid, organic acid and other kinds of metabolites. In the early stage of infection, we observed a significant accumulation of phosphate and a decline in glucose and gluconic acid. At 24 hpi, 13-hydroxylinolenic acid (13(S)-HPOT), asparagine, and oleanolic acid accumulated in the treatment group. More importantly, several plant hormones were involved in the response to the interaction. When disease spots appeared, we found that the intermediate products involved in polyunsaturated fatty acid metabolism and IAA were increased in the treatment group at 72 hpi. The KEGG annotation suggested that these DEMs were involved in multiple pathways (Fig. 6C). Although there were no DEMs activated continuously at all three time points, some metabolism pathways affected the whole process. The results showed that some metabolites of polyketide product biosynthesis and carotenoid biosynthesis were involved in the whole interaction process, and the highest number of metabolites was involved in α-linolenic acid metabolism, especially at 72 hpi. When the pathogen adhered to the plant surface but did not infect the plant, the tea plants initiated energy metabolism, such as the pentose phosphate pathway and glycolysis. When the pathogen developed infection structures, the metabolites involved in flavone and flavonol biosynthesis accumulated. When the fungus started sporulation, polyunsaturated fatty acids and tryptophan metabolism were the major induced pathways. Previous studies have shown that polyunsaturated fatty acids, such as α-linolenic acid and linoleic acid, can be activated into hydroperoxides by lipoxygenases. Subsequently, they are catalyzed by various enzymes and used in many pathways. The intermediate metabolites, 13(S)-HPOT and 12-oxo-phytodienoic acid (12-OPDA), exhibited 1.69- and 9.41-fold changes at 72 hpi, respectively (Fig. 5B). Finally, JA was generated, which is a vital phytohormone in the response to biotic and abiotic stresses in plants^16,28,29^. Tryptophan metabolism is one of the pathways to IAA biosynthesis. IAA accumulated at 72 hpi, and the IAA content in the treatment group increased by 3.82 times compared to that in the control group. Some studies have suggested that auxin can modulate other kinds of hormones in the induction of plant immune responses^14,30^.

### Hormone accumulation at the late stage of the interaction

**Figure 7:**
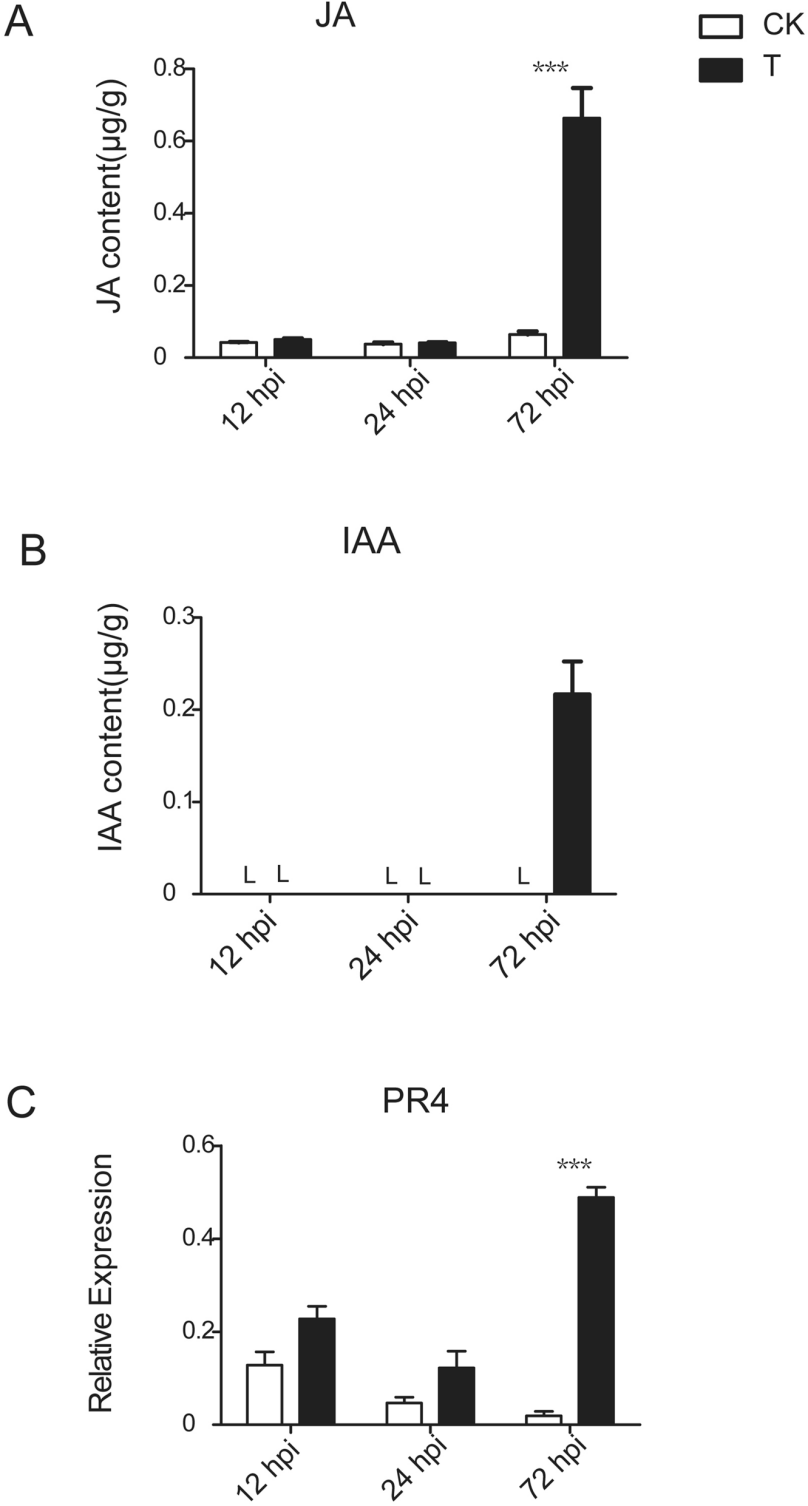
Hormone content determination and qRT-PCR analysis of marker genes of JA-induced defense pathways. (**A**) JA content determination. (**B**) IAA content determination. (**C**) Relative expression of *PR4* gene. L indicates a content lower than the limit of detection. Bars represent ± SE. ***P < 0.001 by the LSD test.

The concentration of plant hormones was quantified by UPLC-MS/MS (Fig. 7A,B). The results showed that the JA and IAA contents were low at 12 hpi and 24 hpi in the control and treatment groups, although the JA and IAA content was significantly increased in the diseased leaves at 72 hpi, suggesting that JA and IAA accumulate in the late stage of the interaction.

To examine the variation in JA signaling system-activated defense genes, the relative expression of *pathogenesis-related protein 4 (PR4)* was subjected to a qRT-PCR analysis against the control groups and the treatment groups during different stages of the tea plant-*Colletotrichum* interaction (Fig. 7). The results showed that the expression level of *PR4* was highly upregulated in infected tea leaves at 72 hpi. The results of the JA content and related gene expression indicated that the JA signaling system plays a crucial role in tea plant-*Colletotrichum* interaction.

### Validation of RNA-seq data

In this study, several genes were selected from the JA/ET biosynthesis pathways and callose deposition development to explore expression profiles by qRT-PCR at different stages postinoculation. The results showed that the qRT-PCR and RNA-seq data had similar expression patterns (Supplementary Fig. S1).

## Discussion

In tea plants, research on the host–pathogen system and the response mechanism of interaction are limited. We used LJ43, a susceptible cultivar of tea plant, and the pathogenic fungus *C. camelliae* to explore the host-fungi interaction system. This study provides information regarding tea plants and *C. camelliae* during the processes from fungal colonization to reproduction (from 12 to 72 hpi). In the present study, we clearly observed the developmental phases of the fungal infection from colonization to reproduction (Fig. 1). The microscopic observation showed that fungal germination, appressoria melanization and sporulation development appeared at 12 hpi, 24 hpi and 72 hpi, respectively, which is similar to the developmental process of other species of *Colletotrichum* while interacting with their hosts. When tea plants were infected with *C. camelliae*, cell wall strengthening and plant hormone signal transduction were stimulated.

Plants can reinforce the cell wall to resist fungal attacks, and modulate their physical structures and chemical components in response to microorganism attacks. The existing research suggests that callose deposition stimulated by PAMPs is a marker for PAMP-triggered immunity (PTI), which is an effective strategy to prevent invasion^22,31^. Our experiments revealed that callose deposition on the host surface coincided with appressoria appearance. As shown in Fig. 4, callose deposition was observed at the infection site. A similar phenomenon occurs in the sorghum-*C. sublineolum* interaction^32^. The GO analysis of the transcriptome showed that the upregulated genes at 24 and 72 hpi were enriched in the defense response by callose deposition but not enriched in this pathway at 12 hpi. These results suggest that callose accumulation is induced by fungal penetration. This evidence was strengthened by the modulation of genes related to callose formation. *CYP83B1* is involved in indolic glucosinolate (IGS) biosynthesis and is required for callose deposition in *Arabidopsis*^33^. *FLS2* is a pattern recognition receptor (PRR) located in the plasma membrane that perceives PAMPs, and the *FLS2-*overexpressing line of *Arabidopsis* exhibits strong callose deposition^34,35^. We found that genes homologous to *FLS2* were highly expressed during infection, which may induce callose deposition. *PEN3* was also induced in the interaction process. *PEN3* is a pleiotropic drug resistance (PDR) protein belonging to the ATP-binding cassette (ABC) transporter superfamily, and it is involved in plant resistance^36^. *pen3* mutant plants have reduced callose deposition^37^. Glycosyl transferases (UGTs) were used to catalyze glycosylation, which is usually accompanied by the deactivation of toxic substances by cytochrome P450s^38^. Vitamin C defective 1 (VTC1) genes are ROS-scavenging genes, and VTC1 *Arabidopsis* mutants exhibited a blockage of callose generation^39^. Chitin elicitor receptor kinase 1 (CERK1) plays dual roles in chitin signaling in rice and *Arabidopsis* defense responses. *cerk1* mutant can reduce callose deposition in rice and *Arabidopsis*^40,41^. We found that those genes implicated in callose accumulation were induced after appressoria development. Notably, the degree of related gene expression was further increased at 72 hpi. A possible reason is that there were more pathogens develop infection structures over time, and thus, relatively stronger gene expression was induced. According to our results, callose deposition may play a positive role in the defense to *C. camelliae* in tea plants, especially, when appressoria develops.

Phytohormones are mediators of the plant stress response. In the present study, we found that the plant hormones JA, IAA and ET had a complex hormonal interplay during the tea plant-*C. camelliae* interaction. Pathways related to ET signaling were enriched at 24 hpi and 72 hpi. Genes involved in ET and JA biosynthesis exhibited a high fold change, especially in the disease stage. According to the number of metabolites in several fatty acid and tryptophan metabolism pathways and the contents of JA and IAA, these hormones seemed to be important in the interaction between the tea plant and *C. camelliae*.

Current studies largely suggest that SA is typically involved in the defense against biotrophs^42^ and that the JA and ET signaling systems play an important role in the regulation of immune responses against necrotrophic fungi^43^. Shi et al. compared healthy tea leaves and leaves infected with *Colletotrichum* and found that SA signaling had a key function in the response of tea plants to *Colletotrichum*^25^. However, we found that SA signaling-related genes were downregulated (Fig. 2B). Shi et al. collected samples from whole tea plants in the field and focused on systemic acquired resistance, which is different from the focus of our study. Many factors affect disease resistance of host plant, therefore, the function of SA in disease resistance needs further study. In the present study, the JA content and expression level of its downstream defense genes were highly increased in infected tea leaves when scabs appeared. We hypothesized that JA signaling may be more important than SA signaling in the tea plant response to *Colletotrichum*, especially in the disease stage.

Hormone signaling crosstalk plays a crucial role in the interaction of plants and pathogens. JA and ET signaling crosstalk is believed to be involved in the plant stress response. In the present study, the JA and ET signaling systems of tea plants showed a notable response through the upregulation of JA/ET pathway-related genes. SAM-1, ACS and ACO, which are related to ET biosynthesis, and AOS and AOC exhibited a significantly increase in expression at 72 hpi when disease spots appeared (Fig. 5A,B). The homologs of *13-LOX* had two different expression profiles, although two of them were notably upregulated at 72 hpi. The intermediate metabolites, such as 13(S)-HPOT and 12-OPDA, significantly increased at 72 hpi. However, few reports have focused on IAA signaling transduction during tea plant stress response. Auxin is considered a signaling molecule involved in the modulation of SA, JA and ET signaling pathways and in the plant disease response^44^. During pathogen infection, the level of endogenous IAA in plants was elevated^45^. The results of the metabolome and content determination indicated that IAA accumulation was induced in the late stage of fungal infection. TFs are also involved in plant defense responses, which transcriptionally modify gene expression. ET response factors (EFRs) are the major downstream regulatory factors of ET signaling pathways and can be activated by the collaboration of the JA and ET pathways^42^. EIN3, another TF, is believed to induce EFR gene expression^46^. MYC2 is a central TF that mediates JA-responsive gene expression and can enhance gene expression associated with defense^29^. Most of the genes encoding EIN3, ERF1, and MYC2 were significantly upregulated when brown disease spots developed. A previous study suggested that APF9 suppressed biotroph resistance, while ARF9 positively enhanced necrotroph resistance. Our results showed that the APF9 DEG was significantly decreased. In summary, we hypothesized that the JA, IAA and ET signaling pathways are induced during pathogen infection and that the multiphytohormone signaling system plays a vital role in the response of tea plant to *Colletotrichum*.

This work used combined transcriptomic and metabolomic analyses of the response of tea plants to the pathogen *C. camelliae*. Host defense reactions were further identified by transcriptomic and metabolomic analyses. Our results demonstrate that callose deposition and the multiphytohormone signaling system play roles in the response of tea plants to the pathogen. These results improve our understanding of the strategies of tea plants in response to fungal attack and may provide available suggestions to facilitate tea plant resistance breeding. Further studies should be carried out for the specific molecular mechanisms of hormone interplay during the interaction of the tea plant with *C. camelliae*. Furthermore, future explorations of the disease resistance mechanism need to focus on the resistance differences among different varieties.

## Materials and methods

### Plant material and *C. camelliae* infection

Tea plant (LJ43) cuttings were collected from experimental gardens in Hangzhou, Zhejiang Province, China. The cuttings were cultivated in 1/2 MS fluid medium at 25 °C. *C. camelliae* (isolate LS_19)^2^ was cultured in potato dextrose broth (PDB) medium at 200 rpm and 25 °C for 3 days. Conidia were harvested by centrifugation at 8,000 rpm in PDB medium and washed with distilled water. The treatment groups were inoculated with a conidial suspension (10^6^ spores/mL) containing 0.1% Tween 20. The control group was inoculated with sterile water. After the inoculation, all samples were wrapped in clean plastic bags to ensure high humidity and promote conidial germination. Each treatment was performed in six biological replicates numbered 1 to 6. Each replicate contained 10 cuttings with independent incubation. One bud and three leaves from the tea plant cuttings were collected at 12, 24, and 72 h postinoculation (hpi) for both the mock and treatment group. The collected samples were frozen in liquid nitrogen first and then stored at − 80 °C for the further analyses. The replicates 1 and replicates 2 were combined by equal quantity for transcriptomic analysis, as were replicates 3 and 4, replicates 5 and 6. All the six biological replicates were subjected to a metabolomics analysis.

### Microscopic and confocal observations

The fungus and callose staining was visualized as previously described^7^. To visualize fungal colonization on host tissues, the infected tea leaves were cut into small pieces (approximately 0.5 mm × 0.5 mm), destained with ethanol-chloroform (3:1, v/v), and stained with 0.025% (w/v) aniline blue in lactophenol. Then, the samples were examined under a Nikon 80i microscope (Japan). The callose measurements were performed with minor modifications. Briefly, the samples were destained with methanol-chloroform (1:2, v/v) and stained with 1% (w/v) aniline blue in 0.15 M K_2_HPO_4_. Then, the callose deposits were observed with under a confocal laser scanning microscope.

### RNA sequencing and data analysis

The total RNA was extracted from the snap-frozen leaves using the RNAprep Pure Plant Kit (Polysaccharides & Polyphenolics-rich) (TIANGEN) according to the manufacturer’s instructions. The total RNA was used to construct a sequencing library using a NEB RNA Library Prep Kit. Then, the library was sequenced on an Illumina platform, and 125 bp/150 bp paired-end reads were generated. Clean data, which were the basis of the subsequent, analysis were obtained by removing adapters, and low quality reads from the raw data, the Q20, Q30 and GC content the in clean data was calculated. The resulting reads were annotated to *Ca. sinensis* var. *sinensis* (https://tpia.teaplant.org/)^47^. Hisat2 v2.0.5 was used to build an index of the reference genome and align the paired-end clean reads^48^. For hisat2, the default parameter was used to map the clean reads to reference genome. StringTie was used for novel gene prediction^49^. The tea plant transcripts were annotated based on the Arabidopsis Information Resource (TAIR) database (https://www.arabidopsis.org/)^27^ with a threshold of E-value < 1e^−5^ by BlastX. The RNA-Seq raw data of the tea plants were uploaded to the NCBI Sequencing Read Archive database and can be accessed with the following accession numbers: SRR10807066, SRR10807067, SRR10807068, SRR10807069, SRR10807070, SRR10807071, SRR10807072, SRR10807073, SRR10807074, SRR10807075, SRR10807076, SRR10807077, SRR10807078, SRR10807079, SRR10807080, SRR10807081, SRR10807082 and SRR10807083.

FeatureCounts was used to count the reads numbers mapped to each gene^50^. FPKM (Fragments Per Kilobase of transcript per Million mapped reads) of each gene were calculated based on the length of the gene and read counts mapped to this gene. The differential expression analysis of the treatment/control groups was performed using the DESeq2 R package^51^. P values were adjusted using Benjamini and Hochberg’s approach. The filtering parameter in DESeq2 was 0.05. Genes were considered DEGs if they had an absolute fold change ≥ 1.5 and a P-value < 0.05. Gene Ontology (GO) enrichment analysis was performed using the Gene Ontology Enrichment Analysis Software Toolkit (GOEAST), and GO terms were considered significantly enriched at P < 0.05^52^. The Venn diagram and heat map were plotted by TBtools software^53^. MapMan software was used to visualize the omics data^54^.

### Metabolomic analysis

The tea leaves were preserved in liquid nitrogen and ground into a powder. Then, 100 mg freeze-dried sample powder were extracted at 4 °C with 0.5 mL 80% methanol solution containing 0.1% methanoic acid. After a 5-min ice bath, the sample was centrifuged at 4 °C and 15,000×*g* for 10 min, and then the water was removed and replaced with 60% methanol. The LC–MS/MS analyses were performed using a Vanquish UHPLC system (THERMO FISHER) coupled with an Orbitrap Q Exactive HF-X mass spectrometer (THERMO FISHER). The samples were injected into a Thermo Hyperil Gold column (C18) operating at a flow rate of 0.2 mL/min and temperature of 40 °C, and Phase A was 5 mM ammonium acetate (pH 9.0), while phase B was methanol. The following gradient was performed to separate the compounds: 98:2 Phase A/Phase B at 0 min, 98:2 Phase A/Phase B at 1.5 min, 0:100 Phase A/Phase B at 12 min, 0:100 Phase A/Phase B at 14 min, 98:2 Phase A/Phase B at 14.1 min, and 98:2 Phase A/Phase B at 16 min. The effluent was connected to electron spray ionization mass spectrometry. The parameters were as follows: spray voltage: 3.2 kV, sheath gas flow rate: 35arb, aux gas flow rate: 10arb, and capillary temperature: 320 °C. The range of m/z was set between 70 and 1,050.

Compound Discoverer 3.0 (CD 3.0, THERMO FISHER) was used to process the raw data. The mzCloud (https://www.mzcloud.org/) and ChemSpider (https://www.chemspider.com/) databases were used to obtain accurate qualitative and relative quantitative results^55^. The significantly different metabolites were identified by an absolute fold change ≥ 1.5 and thresholds of variable importance in projection (VIP) ≥ 1. The Kyoto Encyclopedia of Genes and Genomes (KEGG) (https://www.genome.jp/kegg/) database was used to identify the pathways of the differential metabolites^56^. Principal component analyses (PCAs) were performed using metaX^57^.

### Quantitative real-time PCR

To validate the RNA-seq results, qRT-PCR was conducted using cDNA synthesized from the RNA used for the RNA-seq. The total RNA (1 μg) was used in the cDNA reactions with at PrimeScript 1st Strand cDNA Synthesis Kit (TAKARA). The reaction mixture preparation and PCR amplification were performed as previously described^2^. The primers are listed in Supplementary Table S6. The polypyrimidine tract-binding protein (*CsPTB1*) gene was used as a reference gene^58^. The relative expression was computed using the 2^−ΔCt^ method^59^.

### Quantification of hormone contents

The phytohormone extraction method described by Li et al.^26^ was followed with minor revisions. The samples used to quantify the hormone content were the same batch of metabolomic samples. One hundred milligrams of frozen tea leaf powder from each sample were homogenized in 1 mL ethyl acetate. After centrifugation (12,000 rpm for 20 min at 4 °C), 0.8 mL of the supernatant were transferred to fresh 2 mL tubes. Then, the liquid portion was evaporated using a vacuum concentrator. The residues were resuspended in 0.5 mL of 70% methanol. After centrifugation (12,000 rpm for 10 min at 4 °C), the total supernatant was filtered through a 0.22 μm filter membrane and transferred to glass vials and analyzed by UPLC-MS/MS.

The measurements were conducted using an UPLC/Quattra Premier XE system (WATERS), and 5 μL of each sample were injected into an Acquity UPLC HSS T3 column (100 mm × 2.1 mm, 1.8 μm) operating a flow rate of 0.25 mL/min and 40 °C. The mobile phase, which was composed of solvent A (0.1% formic acid in methanol) and solvent B (0.1% formic acid in water), was used in the gradient mode for the separation. The MS conditions were as follows: spray voltage: 3,000 V, ion source temperature: 120 °C, desolvation temperature: 350 °C, gone gas flow: 50 L/h, desolvation gas flow: 700 L/h, collision gas flow: 0.30 mL/min, and multiplier voltage: 750 V. Mixed standard solutions were prepared to quantify the content of each hormone in the samples. Matrix calibration curves were prepared with concentrations of 1.60 mg/L, 0.32 mg/L, and 0.064 mg/L of sample A. The content was quantified by the external standard method.

### Statistical analysis

The experimental data were subjected to an analysis of variance (ANOVA) using the SPSS 18 software (IBM). The mean values were compared by the least significant difference (LSD) method, and P < 0.05 was considered indicative of a significant difference.

## Supplementary information

Supplementary Information

Supplementary Table S2.

Supplementary Table S3.

Supplementary Table S5.
