# Supplementary material for: Integrated transcriptomic and metabolomic analyses reveal the effects of callose deposition and multihormone signal transduction pathways on the tea plant-Colletotrichum camelliae interaction
Source: Sci Rep. 2020 Jul 30;10:12858. doi: 10.1038/s41598-020-69729-x (PMC7393116; doi:10.1038/s41598-020-69729-x)
Supplement: Supplementary file 1 — Supplementary Information [file 41598_2020_69729_MOESM1_ESM.pdf]

Supplementary Information

Integrated transcriptomic and metabolomic analyses reveal the effects of callose deposition and multihormone signal transduction pathways on the tea plant-*Colletotrichum camelliae* interaction

Qinhua Lu[1], Yuchun Wang[1], Fei Xiong[1, 2], Xinzhong Zhang[1], Nana Li[1], Lu Wang[1], Xinyuan Hao[1], Jianming Zeng[1], Yajun Yang[1]* and Xinchao Wang[1]*

1 Tea Research Institute of Chinese Academy of Agricultural Sciences, Hangzhou, China

2 Tea Research Institute, Nanjing Agricultural University, Nanjing, China

Supplementary Table S1 The quality of RNA-seq

sample	Raw reads	Clean reads	Clean bases(G)	Error rate	Q20	Q30	GC %	Total map
T12h_1	49815466	49071720	7.36	0.03	97.48	93.02	43.52	44601778 (90.89%)
T12h_2	50831572	50251972	7.54	0.03	97.26	92.52	43.49	45525862 (90.6%)
T12h_3	56809808	56148184	8.42	0.03	97.6	93.32	43.66	51151888 (91.1%)
C12h_1	55864712	55334744	8.3	0.03	97.6	93.28	43.94	50593522 (91.43%)
C12h_2	45645770	45095710	6.76	0.03	97.25	92.51	43.73	40942481 (90.79%)
C12h_3	52341694	51820590	7.77	0.03	97.49	93.04	44.08	47385295 (91.44%)
T24h_1	51352088	50779830	7.62	0.03	97.22	92.48	44.11	45981203 (90.55%)
T24h_2	49478520	48820672	7.32	0.03	97.36	92.78	43.97	44309471 (90.76%)
T24h_3	56215976	55542038	8.33	0.03	97.15	92.25	44.24	50531894 (90.98%)
C24h_1	48074970	47315864	7.1	0.03	97.42	92.87	44.24	43099261 (91.09%)
C24h_2	49773122	49051488	7.36	0.03	97.58	93.23	44.11	44694606 (91.12%)
C24h_3	52282844	51442668	7.72	0.03	97.54	93.19	44.27	46805276 (90.99%)
T72h_1	47426446	46557026	6.98	0.03	97.14	92.28	44.47	40140793 (86.22%)
T72h_2	50975512	50026170	7.5	0.03	97.4	92.9	44.04	44201743 (88.36%)
T72h_3	48841340	48073218	7.21	0.03	97.22	92.44	44.31	42628791 (88.67%)
C72h_1	51330228	50127660	7.52	0.03	97.37	92.77	43.8	45542894 (90.85%)
C72h_2	71651004	70494396	10.57	0.03	97.71	93.74	44.03	64269666 (91.17%)
C72h_3	61622244	60832450	9.12	0.03	97.78	93.87	43.95	55464598 (91.18%)

Supplementary Table S2 Significantly differentially expressed genes (DEGs) in different time points

Supplementary Table S3 List of enriched Gene Ontology terms identified from all DEGs

Supplementary Table S4 The top 35 GO terms are listed based on the number of assigned DEGs and P value

GOID	Ontology	Term	Number in input list (q)	Number in the reference list (m)	q/m (%)	P-value
Upregulated genes (12 hpi)						
GO:0051716	BP	cellular response to stimulus	30	2587	1.16	0.000148
GO:0043207	BP	response to external biotic stimulus	23	1502	1.53	5.47E-05
GO:0051707	BP	response to other organism	23	1502	1.53	5.47E-05
GO:0009725	BP	response to hormone	19	1757	1.08	0.0203
GO:0033554	BP	cellular response to stress	18	1162	1.55	0.00054
GO:0006955	BP	immune response	13	748	1.74	0.00371
GO:0042594	BP	response to starvation	11	346	3.18	0.000139
GO:0009620	BP	response to fungus	10	557	1.80	0.0178
GO:0010243	BP	response to organonitrogen compound	9	445	2.02	0.0165
GO:0015706	BP	nitrate transport	6	206	2.91	0.0277
GO:0001666	BP	response to hypoxia	5	100	5.00	0.0101
GO:0017076	MF	purine nucleotide binding	10	605	1.65	0.0283
GO:0030554	MF	adenyl nucleotide binding	10	448	2.23	0.00405
GO:0004601	MF	peroxidase activity	8	100	8.00	1.56E-05
GO:0001067	MF	regulatory region nucleic acid binding	4	21	19.05	0.000494
GO:0051716	BP	cellular response to stimulus	30	2587	1.16	0.000148
Upregulated gene (24 hpi)						
GO:0010243	BP	response to organonitrogen compound	74	445	16.63	1.18E-34
GO:0051716	BP	cellular response to stimulus	181	2587	7.00	1.8E-32
GO:0043207	BP	response to external biotic stimulus	124	1502	8.26	1.43E-27
GO:0051707	BP	response to other organism	124	1502	8.26	1.43E-27
GO:0006955	BP	immune response	80	748	10.70	3.84E-24
GO:0009725	BP	response to hormone	125	1757	7.11	5.47E-22
GO:0070887	BP	cellular response to chemical stimulus	105	1312	8.00	5.84E-22
GO:0033554	BP	cellular response to stress	97	1162	8.35	1.81E-21
GO:0009404	BP	toxin metabolic process	40	225	17.78	2.35E-19
GO:0046942	BP	carboxylic acid transport	43	276	15.58	1.35E-18
GO:0006865	BP	amino acid transport	41	252	16.27	2.1E-18
GO:0015711	BP	organic anion transport	43	298	14.43	2.49E-17
GO:0098754	BP	detoxification	37	226	16.37	1.15E-16
GO:0015706	BP	nitrate transport	34	206	16.50	2.13E-15
GO:0044248	BP	cellular catabolic process	101	1535	6.58	3.44E-15
GO:0072657	BP	protein localization to membrane	44	377	11.67	2.5E-14
GO:0080135	BP	regulation of cellular response to stress	44	388	11.34	7.23E-14
GO:0050776	BP	regulation of immune response	45	428	10.51	5.42E-13
GO:0042446	BP	hormone biosynthetic process	61	741	8.23	6.84E-13

GO:0048585	BP	negative regulation of response to stimulus	41	370	11.08	1.52E-12
GO:0042445	BP	hormone metabolic process	64	819	7.81	1.58E-12
GO:2000377	BP	regulation of reactive oxygen species metabolic process	30	207	14.49	5.03E-12
GO:0051193	BP	regulation of cofactor metabolic process	30	209	14.35	6.49E-12
GO:0009651	BP	response to salt stress	61	785	7.77	8.32E-12
GO:0009723	BP	response to ethylene	39	355	10.99	8.47E-12
GO:0009617	BP	response to bacterium	52	607	8.57	1.47E-11
GO:0016310	BP	phosphorylation	64	868	7.37	2.05E-11
GO:0052542	BP	defense response by callose deposition	17	62	27.42	3.71E-11
GO:0034976	BP	response to endoplasmic reticulum stress	38	358	10.61	4.94E-11
GO:0009595	BP	detection of biotic stimulus	21	106	19.81	6.37E-11
GO:0031348	BP	negative regulation of defense response	33	278	11.87	7.84E-11
GO:0009620	BP	response to fungus	48	557	8.62	9.96E-11
GO:0006970	BP	response to osmotic stress	61	841	7.25	1.47E-10
GO:0071944	CC	cell periphery	176	3735	4.71	6.98E-13
GO:0004601	MF	peroxidase activity	21	100	21.00	1.97E-11
Upregulated gene (72 hpi)						
GO:0051716	BP	cellular response to stimulus	610	2587	23.58	1.58E-81
GO:0010243	BP	response to organonitrogen compound	208	445	46.74	2.81E-80
GO:0033554	BP	cellular response to stress	347	1162	29.86	6.85E-71
GO:0034976	BP	response to endoplasmic reticulum stress	157	358	43.85	1.91E-55
GO:0006955	BP	immune response	238	748	31.82	3.3E-53
GO:0043207	BP	response to external biotic stimulus	368	1502	24.50	4.28E-51
GO:0051707	BP	response to other organism	368	1502	24.50	4.28E-51
GO:0006865	BP	amino acid transport	116	252	46.03	2.58E-43
GO:0046942	BP	carboxylic acid transport	122	276	44.20	2.71E-43
GO:0015711	BP	organic anion transport	122	298	40.94	4.47E-39
GO:0009404	BP	toxin metabolic process	97	225	43.11	3.78E-33
GO:0098754	BP	detoxification	96	226	42.48	3.67E-32
GO:0009725	BP	response to hormone	356	1757	20.26	2.1E-30
GO:0070887	BP	cellular response to chemical stimulus	287	1312	21.88	7.38E-30
GO:0015833	BP	peptide transport	247	1084	22.79	2.95E-28
GO:0035966	BP	response to topologically incorrect protein	121	370	32.70	1.01E-27
GO:0016310	BP	phosphorylation	210	868	24.19	1.77E-27
GO:0006986	BP	response to unfolded protein	79	188	42.02	5.3E-26
GO:0052542	BP	defense response by callose deposition	43	62	69.35	1.97E-25
GO:0009617	BP	response to bacterium	161	607	26.52	2.01E-25
GO:0072657	BP	protein localization to membrane	118	377	31.30	3.95E-25
GO:0044248	BP	cellular catabolic process	307	1535	20.00	5.7E-25
GO:0006970	BP	response to osmotic stress	199	841	23.66	1.05E-24
GO:0050776	BP	regulation of immune response	127	428	29.67	1.1E-24

GO:0080135	BP	regulation of cellular response to stress	118	388	30.41	6.46E-24
GO:0045184	BP	establishment of protein localization	218	975	22.36	1.11E-23
GO:0009651	BP	response to salt stress	186	785	23.69	4.1E-23
GO:0015824	BP	proline transport	45	74	60.81	4.98E-23
GO:0009723	BP	response to ethylene	109	355	30.70	1.95E-22
GO:0071944	CC	cell periphery	761	3735	20.37	2E-72
GO:0012505	CC	endomembrane system	380	1675	22.69	3.43E-44
GO:0051740	MF	ethylene binding	7	5	140.00	0
GO:1901265	MF	nucleoside phosphate binding	219	839	26.10	8.06E-34
GO:0000166	MF	nucleotide binding	219	839	26.10	8.06E-34
GO:0017076	MF	purine nucleotide binding	155	605	25.62	8.92E-23
Downregulated gene (12 hpi)						
GO:0015979	BP	photosynthesis	21	403	5.21	1.14E-09
GO:0009657	BP	plastid organization	16	406	3.94	9.79E-06
GO:0006733	BP	oxidoreduction coenzyme metabolic process	16	427	3.75	1.76E-05
GO:0006091	BP	generation of precursor metabolites and energy	18	605	2.98	6.19E-05
GO:0044283	BP	small molecule biosynthetic process	28	1384	2.02	8.06E-05
GO:0017144	BP	drug metabolic process	20	831	2.41	0.000261
GO:0070838	BP	divalent metal ion transport	10	210	4.76	0.000385
GO:0051186	BP	cofactor metabolic process	21	935	2.25	0.000387
GO:0006790	BP	sulfur compound metabolic process	18	712	2.53	0.000405
GO:0006732	BP	coenzyme metabolic process	16	585	2.74	0.000537
GO:0071214	BP	cellular response to abiotic stimulus	9	173	5.20	0.000538
GO:0104004	BP	cellular response to environmental stimulus	9	173	5.20	0.000538
GO:0055082	BP	cellular chemical homeostasis	11	308	3.57	0.00136
GO:0019725	BP	cellular homeostasis	11	325	3.38	0.00212
GO:0019637	BP	organophosphate metabolic process	21	1095	1.92	0.00273
GO:0048731	BP	system development	37	2640	1.40	0.00273
GO:0006796	BP	phosphate-containing compound metabolic process	28	1749	1.60	0.003
GO:0043933	BP	protein-containing complex subunit organization	14	546	2.56	0.00304
GO:0022900	BP	electron transport chain	6	93	6.45	0.00457
GO:0006081	BP	cellular aldehyde metabolic process	12	431	2.78	0.00473
GO:0048506	BP	regulation of timing of meristematic phase transition	4	30	13.33	0.00504
GO:0048510	BP	regulation of timing of transition from vegetative to reproductive phase	4	30	13.33	0.00504
GO:0006793	BP	phosphorus metabolic process	28	1837	1.52	0.00611
GO:0031323	BP	regulation of cellular metabolic process	34	2462	1.38	0.00669
GO:0048367	BP	shoot system development	21	1241	1.69	0.0114

GO:0034765	BP	regulation of ion transmembrane transport	6	117	5.13	0.0127
GO:0010155	BP	regulation of proton transport	5	77	6.49	0.0145
GO:1904062	BP	regulation of cation transmembrane transport	5	79	6.33	0.0161
GO:0000302	BP	response to reactive oxygen species	9	317	2.84	0.025
GO:0031975	CC	envelope	30	902	3.33	1.14E-09
GO:0044422	CC	organelle part	57	3130	1.82	2.92E-09
GO:0009986	CC	cell surface	3	14	21.43	0.00953
GO:0008187	MF	poly-pyrimidine tract binding	4	19	21.05	0.00106
GO:0008266	MF	poly(U) RNA binding	4	19	21.05	0.00106
GO:0003727	MF	single-stranded RNA binding	4	40	10.00	0.0127
Downregulated genes (24 hpi)						
GO:0006796	BP	phosphate-containing compound metabolic process	165	1749	9.43	4.96E-40
GO:0006793	BP	phosphorus metabolic process	169	1837	9.20	1.05E-39
GO:0016043	BP	cellular component organization	219	2961	7.40	8.85E-38
GO:0044237	BP	cellular metabolic process	398	7935	5.02	1.7E-33
GO:0009657	BP	plastid organization	72	406	17.73	5.12E-33
GO:0006081	BP	cellular aldehyde metabolic process	74	431	17.17	5.14E-33
GO:0006996	BP	organelle organization	162	1947	8.32	1.43E-32
GO:0015979	BP	photosynthesis	71	403	17.62	2.29E-32
GO:0005975	BP	carbohydrate metabolic process	120	1281	9.37	6.34E-28
GO:0019637	BP	organophosphate metabolic process	106	1095	9.68	1.44E-25
GO:0006790	BP	sulfur compound metabolic process	82	712	11.52	2.32E-24
GO:0006091	BP	generation of precursor metabolites and energy	74	605	12.23	1.75E-23
GO:0006733	BP	oxidoreduction coenzyme metabolic process	62	427	14.52	1.86E-23
GO:1901564	BP	organonitrogen compound metabolic process	262	4863	5.39	2.06E-23
GO:0051186	BP	cofactor metabolic process	93	935	9.95	3.69E-23
GO:0006732	BP	coenzyme metabolic process	70	585	11.97	1.31E-21
GO:0048367	BP	shoot system development	101	1241	8.14	7.74E-19
GO:0006725	BP	cellular aromatic compound metabolic process	192	3385	5.67	1.79E-18
GO:0043933	BP	protein-containing complex subunit organization	61	546	11.17	2.64E-17
GO:0016310	BP	phosphorylation	78	868	8.99	9.94E-17
GO:0009311	BP	oligosaccharide metabolic process	37	210	17.62	1.31E-16
GO:0046483	BP	heterocycle metabolic process	174	3083	5.64	2.66E-16
GO:1901576	BP	organic substance biosynthetic process	247	5060	4.88	3.44E-16
GO:0000226	BP	microtubule cytoskeleton organization	38	238	15.97	1.35E-15
GO:0090698	BP	post-embryonic plant morphogenesis	56	511	10.96	1.68E-15
GO:0010073	BP	meristem maintenance	41	284	14.44	2.76E-15

GO:0009658	BP	chloroplast organization	38	251	15.14	7.74E-15
GO:0009653	BP	anatomical structure morphogenesis	116	1769	6.56	9.73E-15
GO:0044283	BP	small molecule biosynthetic process	98	1384	7.08	2.42E-14
GO:0043085	BP	positive regulation of catalytic activity	25	115	21.74	2.62E-13
GO:0044422	CC	organelle part	199	3130	6.36	4.47E-25
GO:0071944	CC	cell periphery	203	3735	5.44	1.31E-17
GO:0031975	CC	envelope	76	902	8.43	8.66E-15
GO:0030554	MF	adenyl nucleotide binding	57	448	12.72	9.32E-19
GO:0017076	MF	purine nucleotide binding	60	605	9.92	1.14E-14
Downregulated gene (72 hpi)						
GO:0009657	BP	plastid organization	175	406	43.10	1.21E-76
GO:0006081	BP	cellular aldehyde metabolic process	179	431	41.53	2.79E-75
GO:0006796	BP	phosphate-containing compound metabolic process	395	1749	22.58	4.17E-74
GO:0006793	BP	phosphorus metabolic process	405	1837	22.05	6.81E-73
GO:0006996	BP	organelle organization	417	1947	21.42	2.71E-71
GO:0016043	BP	cellular component organization	536	2961	18.10	3.21E-66
GO:0051186	BP	cofactor metabolic process	258	935	27.59	3.45E-65
GO:0044237	BP	cellular metabolic process	1063	7935	13.40	2E-63
GO:0019637	BP	organophosphate metabolic process	269	1095	24.57	9.58E-57
GO:0015979	BP	photosynthesis	142	403	35.24	7.12E-49
GO:0046483	BP	heterocycle metabolic process	498	3083	16.15	2.08E-45
GO:0006725	BP	cellular aromatic compound metabolic process	531	3385	15.69	6.29E-45
GO:0009658	BP	chloroplast organization	103	251	41.04	3.71E-42
GO:0006733	BP	oxidoreduction coenzyme metabolic process	135	427	31.62	2.38E-40
GO:0061024	BP	membrane organization	92	214	42.99	1.38E-39
GO:1901564	BP	organonitrogen compound metabolic process	671	4863	13.80	1.07E-38
GO:0048367	BP	shoot system development	254	1241	20.47	3.37E-38
GO:0005975	BP	carbohydrate metabolic process	257	1281	20.06	4.12E-37
GO:0006732	BP	coenzyme metabolic process	154	585	26.32	1.91E-35
GO:0006790	BP	sulfur compound metabolic process	172	712	24.16	1.51E-34
GO:0009311	BP	oligosaccharide metabolic process	81	210	38.57	1.01E-30
GO:1901576	BP	organic substance biosynthetic process	662	5060	13.08	1.08E-30
GO:0051656	BP	establishment of organelle localization	57	107	53.27	1.72E-30
GO:0006091	BP	generation of precursor metabolites and energy	147	605	24.30	1.16E-29
GO:0048731	BP	system development	401	2640	15.19	1.35E-29
GO:0007275	BP	multicellular organism development	489	3539	13.82	1.02E-26
GO:0009791	BP	post-embryonic development	338	2169	15.58	1.54E-26
GO:0090698	BP	post-embryonic plant morphogenesis	126	511	24.66	5.77E-26
GO:0044422	CC	organelle part	572	3130	18.27	5.79E-73

GO:0044464	CC	cell part	2107	21711	9.70	5.92E-29
GO:0031975	CC	envelope	186	902	20.62	5.63E-28
GO:0000166	MF	nucleotide binding	184	839	21.93	3.69E-31
GO:1901265	MF	nucleoside phosphate binding	184	839	21.93	3.69E-31
GO:0030554	MF	adenyl nucleotide binding	122	448	27.23	2.1E-29
GO:0017076	MF	purine nucleotide binding	146	605	24.13	3.96E-29

Supplementary Table S5 List of the differentially expressed metabolites (DEMs) in different time points

Supplementary Table S6 qRT-PCR validation primers

Gene ID	Primer	Sequence (5’→3’)
TEA026913	Forward（F）	TCGGGTTTCTTCATCCCTAGAC
	Reverse（R）	CGTACCATTGGACAGTAGTGGA
novel.17836	F	ATGCACTCGAAGGACCAATC
	R	GTCGACTAAGTTGTGCCATACC
TEA012289	F	GCAAGCGACCAAGTTTATGG
	R	CTCTCTCTCTACTCGTCTGATCTC
TEA025907	F	CCACATGGTTGAGCCTAGAA
	R	CAAGATCCGCATGGTTCTCT
TEA019216	F	GAGGCGCTCTGTAACATCTA
	R	CCAAGGAAGTGGTCAGTGTA
TEA001041	F	GAAACGGGACATGGTGATAGAG
	R	CAGAACGTGCCTCAACATCT
TEA030190	F	CGGCTGATAAGGCAAAGGAA
	R	ACTCGAGGTAGTGGGAATCA
TEA026494	F	GACAGGTCATCCAGAGACAATC
	R	CTGGAACACCAAGTCCTGATAG
TEA011776	F	CGGTAGGTCATGGTGACAAG
	R	TTGGTTCGAGCAATGGTAGG
TEA004708	F	GAAACTGATGCAGGGCTTCT
	R	GGGATGAACCATGGCTAACA

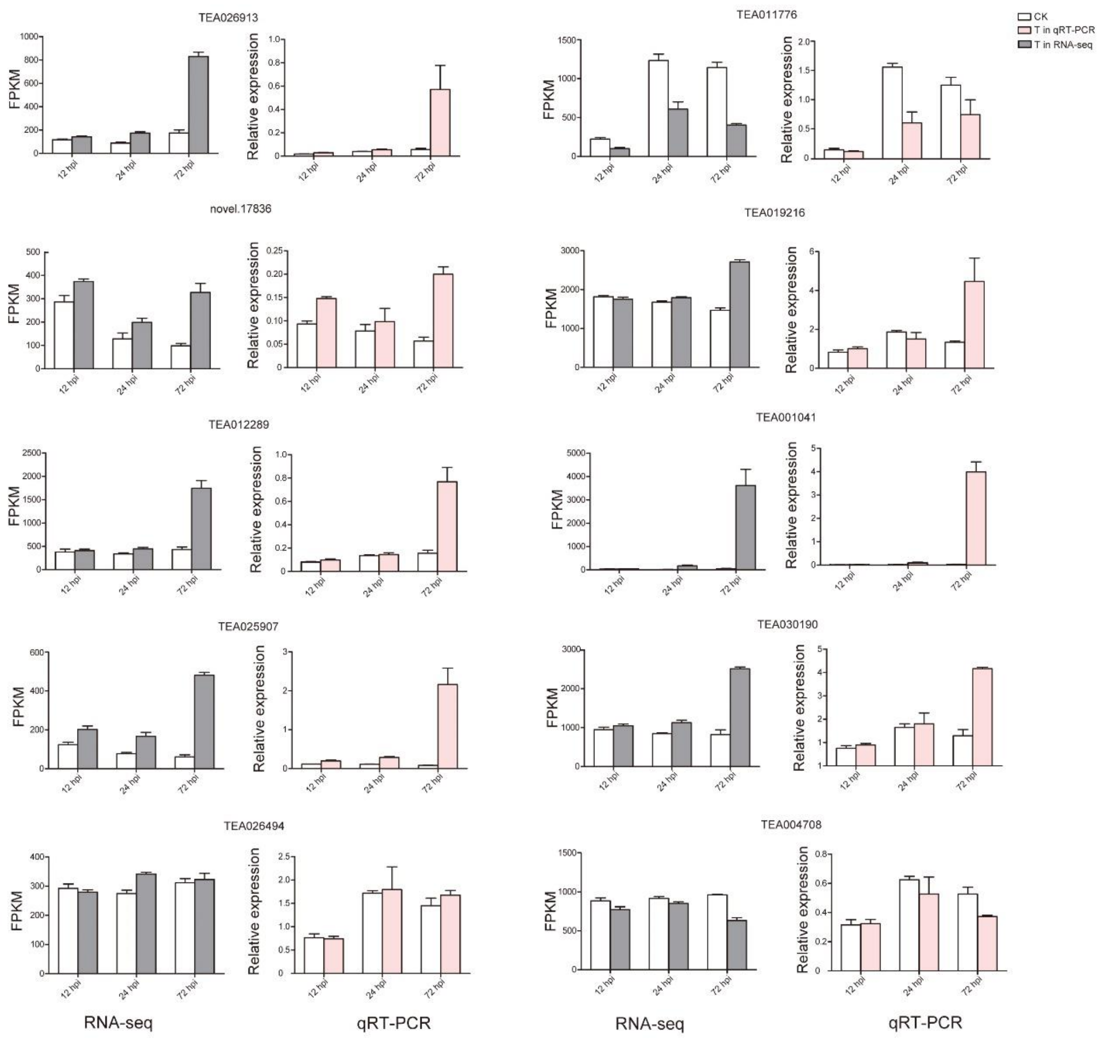

Figure S1 Expression profile comparisons between the RNA-Seq and qRT-PCR data
